# Longitudinal changes in sports activity from pre-diagnosis to first five years post-diagnosis: a prospective Chinese breast cancer cohort study

**DOI:** 10.1186/s12885-020-07517-6

**Published:** 2020-10-19

**Authors:** Yuan-Yuan Lei, Suzanne C. Ho, Carol Kwok, Ashley Cheng, Ka Li Cheung, Roselle Lee, Winnie Yeo

**Affiliations:** 1 Department of Clinical Oncology, Prince of Wales Hospital, the Chinese University of Hong Kong, Shatin, New Territories, Hong Kong SAR, China; 2 Division of Epidemiology, the Jockey Club School of Public Health and Primary Care, the Chinese University of Hong Kong, New Territories, Hong Kong SAR, China; 3 Department of Clinical Oncology, Princess Margaret Hospital, Hong Kong SAR, China; 4 Hong Kong Cancer Institute, State Key Laboratory in Oncology in South China, Faculty of Medicine, the Chinese University of Hong Kong, New Territories, Hong Kong SAR, China

**Keywords:** Breast cancer, Exercise, Physical activity, Pre- and post-diagnosis, Chinese women

## Abstract

**Background:**

To compare change in level of physical activity between pre-and post- diagnosis of breast cancer in Chinese women.

**Methods:**

Based on an on-going prospective study consisting of a sample of Chinese women with breast cancer, a validated modified Chinese Baecke questionnaire was used to measure physical activity at baseline (12 months before cancer diagnosis), 18-, 36- and 60-months after diagnosis (over the previous 12 months before each interview).

**Results:**

In our cohort of 1462 Chinese women with a mean age of 52 years, the mean level of physical activity at post-diagnosis was 9.6 metabolic equivalent of task (MET)-hours/week, which was significantly higher than that at pre-diagnosis with mean level of 5.9 MET-hours/week (*P* < 0.001). The mean levels of physical activity at 18-, 36- and 60-months follow-up were 9.9, 9.8 and 9.3 MET-hours/week, respectively. There was no significant difference between any two of the three follow-ups at post-diagnosis. The proportions of participant who met World Cancer Research Fund/ American Institute for Cancer Research (WCRF/AICR) recommendation before and after cancer diagnosis were both low, being 20.7 and 35.1%, respectively. Compared to pre-diagnosis, most of the patients improved or had no change on level of physical activity at post-diagnosis, with the respective proportion being 48.2 and 43.8%.

**Conclusions:**

Adherence to current lifestyle recommendation for cancer survivors, Chinese women with breast cancer significantly increased level of physical activity level after cancer diagnosis, and such improvement was sustained to 5 years post-diagnosis. The proportion of patients who met the exercise recommendation for cancer survivors was still low. Encouraging patients on the importance of durable high level of physical activity in breast cancer survivorship is warranted.

## Background

In the United States, breast cancer is the most common cancer among females, accounting for 30% of all new cancer diagnoses in women [1]. In Hong Kong, breast cancer is also the leading cancer, and more than 80% of newly diagnosed patients have early stage breast cancer [2]. Surgical treatment is the major maneuver in the management of early stage patients. However, such procedure may lead to breast cancer related lymphedema (BCRL), axillary web syndrome (AWS) and cancer-related fatigue. It has been reported that BCRL involves more than 25% of breast cancer survivors, [3, 4] while axillary web syndrome (AWS) after axillary lymph node dissection affects in 6 to 85.4% [5, 6]. Cancer-related fatigue is a common disabling conditions in breast cancer survivors [7]. It has been estimated that approximately one-third of cancer survivors have clinically significant fatigue up to 6 years following treatment [8]. Breast cancer survivorship care has aroused increasing attention in recent years, and the management of the above-mentioned complications are topics of concern in the rehabilitation field. The beneficial effects of physical activity on cancer related complications have been supported by strong evidence. A systematic review which included 29 studies suggested that resistance exercise could potentially alleviate BCRL [9]. Two meta-analyses including high quality studies have shown that supervised aerobic exercise was effective in improving cancer-related fatigue among breast cancer survivors [10, 11].

Current guidelines recommended breast cancer survivors to engage in regular physical activity, aiming for at least 150 min of moderate or 75 min of vigorous aerobic exercise per week [12–14]. However, studies in the US have shown that less than half of breast cancer survivors (37–48%) met the exercise recommendations [15, 16]. The diagnosis of cancer has been considered as a “teachable moment”, when individuals could be motivated to make positive lifestyle changes, including increasing physical activity [17, 18]. Hence, the assessment of exercise habits among cancer survivors at the time of diagnosis and thereafter could be of importance. Healthcare providers many utilize this teachable moment as a chance to educate patients to undergo some lifestyle interventions.

A number of studies have investigated physical activity levels in breast cancer survivors during an immediate period, about 1 year after diagnosis [19–24]. Overall, these studies reported that the level of physical activity was reduced during the immediate period post-diagnosis. Only one prospective study conducted in the US among breast cancer patients has reported the changes of physical activity over a more protracted period, from 2 year before diagnosis to 30-months after diagnosis [25]. Up to now, no study has reported the changes of physical activity before and after breast cancer diagnosis among Asian women including Chinese.

The primary aim of this present study was to compare the changes in level of physical activity from 1 year before diagnosis to 5 years after diagnosis using data from an ongoing prospective cohort of Chinese women with early stage breast cancer. This study also examined the associations between socio-demographic, clinical and lifestyle factors with changes in level of physical activity before and after cancer diagnosis. The hypothesis of the present study was that breast cancer patients would change their level of physical activity after breast cancer diagnosis, and several socio-demographic, clinical and lifestyle factors may be associated with such changes.

## Methods

### Study cohort

The present study was based on a prospective cohort study titled “The Hong Kong NTEC-KWC Breast Cancer Survival Study (HKNKBCSS)”, which was designed to evaluate whether dietary phytoestrogens and other lifestyle factors affect breast cancer patients’ survival outcome [26]. This study recruited participants at two regional public cancer centers (New Territories East and Kowloon West) in Hong Kong. Consecutive breast cancer patients attending two regional cancer centers in Hong Kong, who were potential eligible participators, were invited to participate in this project. All eligible women should have confirmed, newly diagnosed (defined as having diagnosed within 12 months before study entry), stage 0-III breast cancer [27]. Patients who had prior history of breast or other cancers were ineligible. The study was approved by the Joint CUHK-NTEC Clinical Research Ethics Committee and the KWC Research Ethics Committee of the Chinese University of Hong Kong and the Hong Kong Hospital Authority.

In total, 1462 consented patients enrolled in this study between January 2011 and February 2014. Enrolled patients were interviewed at baseline (T0; within 12 month of breast cancer diagnosis), 18 months (T1; conducted between 12 and 24 months after diagnosis), 36 months (T2; conducted between 30 and 42 months) and 60 months (T3; conducted between 54 and 66 months) after breast cancer diagnosis. The four interviews used similar questionnaires to collect data. As of January 2019, the 60-months follow-up interview has been completed.

### Data collection

Trained interviewers carried out the baseline and follow-up assessments. At baseline, detailed personal data was collected using standardized questionnaire, which included socio-demographic characteristics such as age at diagnosis, education level, household income, marital status; menopausal status; medical history including diabetes, cardiovascular diseases; lifestyle factors including dietary intake, physical activity, smoking, drinking and supplement use. Height and weight was measured by standard protocol, and body mass index (BMI) was categorized as following: underweight < 18.5 kg/m^2^, normal 18.5–22.9 kg/m^2^, overweight 23–24.9 kg/m^2^, obese ≥25 kg/m^2^ [28]. At T1, T2 and T3, patients were asked to complete similar questionnaires for collecting dietary intake and physical activity data. Medical records were retrieved for patients’ clinical characteristics and anti-cancer treatment.

### Physical activity measurements

Patients reported physical activity habits in the previous year before cancer diagnosis at T0 assessment. Subsequently, patients recalled habitual physical activity over the previous year during interviews at T1, T2 and T3 assessment. The mean value at T1, T2 and T3 were defined as overall level of physical activity at post-diagnosis.

Physical activity was measured by a validated modified Chinese Baecke questionnaire (Supplementary Table 1), which consisted of physical activity at work, in doing housework, at leisure time (time excluding working and playing sports or exercise) and in doing sports [29]. This study only analyzed physical activity in doing sports for two reasons; firstly, WCRF /AICR recommendations have recommended on sports activity; secondly, data from such an approach would also be more comparable with other studies which have mostly been based on sports and exercises. In each assessment, the subjects who did sport or exercise were asked to specify the activities that they did categorically (up to 2 self-reported sport activities), and the number of hours per week and months of the year they did the activity was recorded. The scores of metabolic equivalent of task (MET)-hours per week was calculated by multiplying the corresponding MET value of the activity by the time (hours per week) engaged in this activity [30]. The MET code for each sport was based on the values in the Ainsworth compendium of physical activity [30]. Summing the score of MET-hours per week of each activity provides the total level of physical activity.

The American Cancer Society (ACS) and World Cancer Research Fund/American Institute for Cancer Research (WCRF/AICR) recommendations for cancer survivors both suggest that individual subject should “be moderately physically active, equivalent to brisk walking, for at least 30 minutes every day.” [13, 14] This recommendation could be operationalized as engaged in moderate or fast walking and/or other moderate or strenuous activity for an average of 30 min per day, for at least 5 days per week. For instance, the scores of MET-hours per week for moderate or fast walking, 30 min per day, 5 days per week was 10 (calculated as follow: 4.0 METs/hour * 0.5 h/day * 5 days/week). According to the level of sports activity, patients were categorized into 3 groups as follow: no exercise (0 MET-hours/week), low-exercise-level (< 10 MET-hours/week) and high-exercise-level (≥10 MET-hours/week). Patients who belonged to high-exercise-level group were those who met the exercise recommendations for both healthy adults [31] and cancer survivors [13, 14]..

The individual change of physical activity between pre-diagnosis (level at T0) and post-diagnosis (mean level at T1, T2 and T3) was classified into three groups: improved (moved from no exercise to low-exercise-level group or from low-exercise-level to high-exercise-level group), no change (kept in the same level of exercise group) and declined (moved from low/high-exercise-level to no exercise group or from high-exercise-level to low-exercise-level group). The absolute change of physical activity between pre-diagnosis and post-diagnosis was calculated by having “the mean level of MET-hours/week at T1, T2 and T3” minus “the level of MET-hours/week at T0”.

### Statistical analysis

The HKNKBCSS was primarily designed to evaluate soy intake and breast cancer prognosis. Based on the assumption that the 5-year mortality rate in the non-exposed or low intake is 20% and the hazard ratio of mortality of 0.7 among the high soy intake relative to the low intake group, [32] this study have recruited 1462 cases and met the target sample size of 1350 to detect the hazard ratio of 0.7 at 5% level of significance and 80% power. During each follow-up, those who have completed the baseline data collection and are free of recurrence at the time of follow-up were invited. Patients who lost one of the follow-ups could also join the next follow-up study. All enrolled patients were actively followed-up, and their recurrence and survival statuses were collected yearly by reviewing medical records or asking patients through telephone call.

The difference in physical activity between pre- and post-diagnosis (mean level at T1, T2 and T3) were examined by paired two-sample t-test. Similar analysis was also conducted to detect difference at any two time-points between T1, T2 and T3 post-diagnosis. Chi-square test was used to compare the frequency of patients who changed from no or low-exercise level to high-exercise level after diagnosis by socio-demographic, clinical and lifestyle factors. Multivariate logistic regression model was used to investigate the odds ratio of such change by socio-demographic, clinical and lifestyle factors. The independent two-sample t-test or one-way ANOVA was used to compare the absolute change of physical activity between pre and post-diagnosis by socio-demographic, clinical and lifestyle factors. Multivariate linear regression model was used to investigate the association of physical activity change between pre- and post-diagnosis with socio-demographic, clinical and lifestyle factors. All analyses were performed using SPSS 21.0; and *P* value < 0.05 at two-sided analysis were considered statistically significant.

## Results

### Participants’ characteristics

**Fig. 1:**
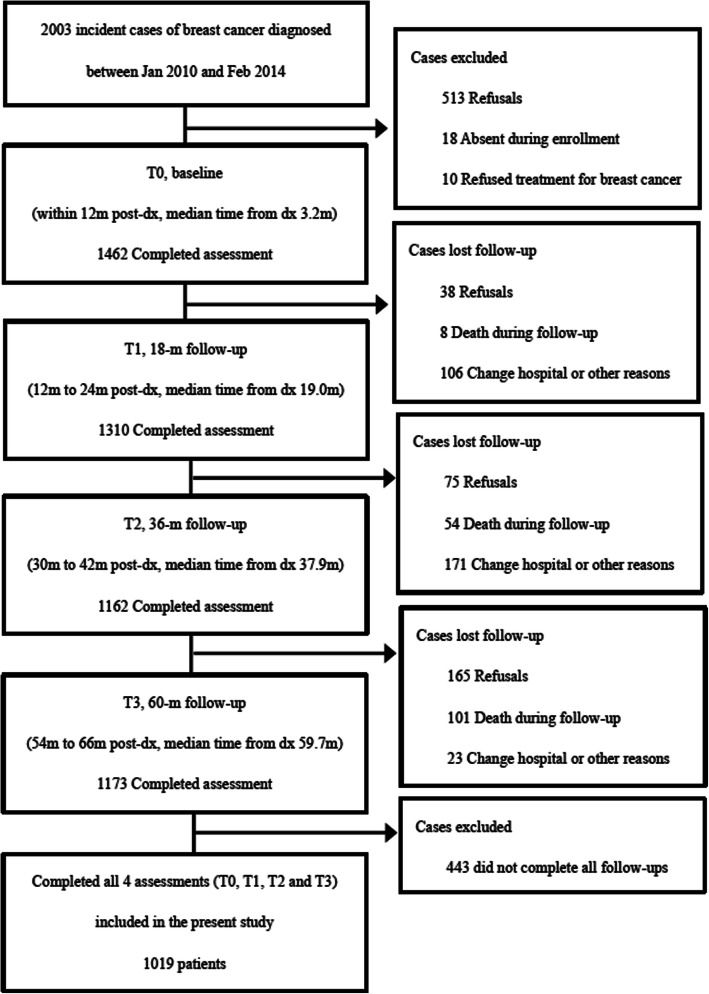
Study flow chart. Abbreviations: m, month; dx, diagnosis

In total, 1462 patients completed baseline interview and enrolled into the study; 1310 (89.6%), 1162 (79.5%) and 1173 (80.2%) participants completed the 18, 36 and 60-months follow-up interviews respectively. Among 1462 enrolled patients, 1019 patients completed all four assessments (T0, T1, T2 and T3) and were included in the present analysis (Fig. 1). Those who did not complete all follow-ups were excluded. Patients who completed all follow-ups at the four time-points showed similar baseline characteristics with the whole cohort.

The baseline demographic and clinical characteristics of patients included in this analysis are provided in Table 1. The mean age at diagnosis was 52 years. Sixty-two percent had no comorbidity. Nearly half of the patients (47%) were post-menopausal at diagnosis. Obesity was observed in 28% of patients. Thirty-seven percent belonged to AJCC stage 0-I, 46% stage II and 17% stage III. ER, PR and HER2 positivity were presented in 75, 59 and 17% of the patients. All patients in this analysis have completed breast cancer surgery. Majority of patients had received adjuvant chemotherapy, radiotherapy and endocrine therapy, with the corresponding figure being 76.0, 70.8 and 76.3%, respectively. At baseline, only a small group of patients were current smokers or drinkers, at 1.1 and 1.5%, respectively.

**Table 1:** Baseline socio-demographic and clinical characteristics of patients included in this study (*n* = 1019)

Characteristics	Number of patients	Frequency, %
Age, mean ± SD, year	52.1 ± 8.8	
Education level		
High school or below	868	85.2
Collage or above	151	14.8
Marital status		
Married or cohabitating	728	71.4
Unmarried or divorced or widowed	291	28.6
Household income (HKD/month)		
< 15,000	463	45.4
15,000-30,000	326	32.0
30,000-50,000	151	14.8
≥ 50,000	79	7.8
Employment status		
Full time	378	37.1
Part time	129	12.7
Not working	512	50.2
Menopausal status		
Pre-menopausal	539	52.9
Post-menopausal	480	47.1
BMI at diagnosis, kg/m^2^		
Underweight (< 18.5)	37	3.6
Normal (18.5–22.9)	488	47.9
Overweight (23–24.9)	209	20.5
Obese (≥25)	285	28.0
Number of comorbidities		
None	629	61.7
1	259	25.4
2	101	9.9
≥ 3	30	2.9
AJCC stage at diagnosis		
0-I	375	36.9
II	471	46.2
III	169	16.6
Missing	4	0.4
Histology		
IDC	858	84.2
ILC	31	3.0
DCIS	56	5.5
Others	74	7.3
ER status		
Positive	763	74.9
Negative	233	22.9
Missing	23	2.3
PR status		
Positive	598	58.7
Negative	395	38.8
Missing	26	2.6
HER 2 status		
Positive	274	26.9
Negative	686	67.3
Missing	59	5.8
Surgery		
Mastectomy	628	61.6
Conservation	391	38.4
Chemotherapy		
Yes	774	76.0
No	245	24.0
Radiotherapy		
Yes	721	70.8
No	298	29.2
Endocrine therapy		
Yes	777	76.3
No	242	23.7
Smoking status		
Yes	11	1.1
No	1008	98.9
Drinking status		
Yes	15	1.5
No	1004	98.5

*Abbreviations*: *SD* standard deviation, *HKD* Hong Kong dollars, *BMI* body mass index, *AJCC* American joint Committee on cancer, *IDC* invasive ductal carcinoma, *ILC* invasive lobular carcinoma, *DCIS* ductal carcinoma in situ, *ER* estrogen receptor, *PR* progesterone receptor, *HER 2* human epidermal-growth-factor receptor 2

### Comparison of physical activity between pre- and post-diagnosis

The mean levels of physical activity at T0 (pre-diagnosis), T1, T2 and T3 were 5.9, 9.9, 9.8 and 9,3 MET-hours/week, respectively. The overall level of physical activity at post-diagnosis was significantly higher than that at pre-diagnosis, with mean values of 9.6 vs 5.9 MET-hours/week respectively, *P* < 0.001 (Table 2). When comparing between T1 and T2, T1 and T3 as well as T2 and T3, there was no significant difference between any two follow-ups at post-diagnosis (*P* > 0.05, each). The proportions of participants who never did exercise at pre- and post-diagnosis were 46.4 and 10.9%, respectively. However, for those did exercise, the proportions of participants who met the exercise recommendation of WCRF/AICR were relatively low; the figures increased from 20.7% at pre-diagnosis to 35.1% post-diagnosis (*P* < 0.001).

**Table 2:** Comparison of level of physical activity across four study time-points

Level of recreational physical activity	T0	T1	T2	T3	Mean of T1,2,3	*P*_T0 vs mean of T1,2,3_
MET-hours/week, mean ± SD	5.9 ± 11.2	9.9 ± 13.0	9.8 ± 14.2	9.3 ± 14.4	9.6 ± 11.6	< 0.001
Physical activity group, n (%)						
No exercise	473 (46.4)	209 (20.5)	298 (29.2)	340 (33.4)	111 (10.9)	
Low-exercise-level group	335 (32.9)	465 (45.6)	397 (39.0)	376 (36.9)	550 (54.0)	
High-exercise-level group	211 (20.7)	345 (33.9)	324 (31.8)	303 (29.7)	358 (35.1)	

*Abbreviations*: *MET* metabolic equivalent of task, *SD* standard deviation

### Change in level of physical activity between pre- and post-diagnosis in individual patient

The changes of physical activity between pre- and post-diagnosis in individual patient are shown in supplementary Table 2. Compared to pre-diagnosis, most of the patients improved or had no change in level of physical activity at post-diagnosis, with the respective proportions being 48.2 and 43.8%, respectively. Only 8.0% of the patients had their level of physical activity declined at post-diagnosis.

In total, 20.7% of patients changed from no or low to high level of physical activity after diagnosis. The proportions patients with such positive change by socio-demographic, clinical and lifestyle factors are presented in Table 3. Univariate analysis showed that patients with the following characteristics are more likely to make such positive changes: patients who were aged≥40 years (compared to those who were aged < 40 years), non-obese (compared to obese), stage II-III (compared to stage 0-I), and having received adjuvant chemotherapy (compared to no chemotherapy).

**Table 3:** Changes from no or low to high level of exercise group for individual patient by baseline socio-demographic and clinical factors

Characteristics	N	%	Changes from no or low to high exercise group, n (%)	*P* value
Total	1019	100.0	211 (20.7)	
Age at diagnosis, year				0.068
< 40	92	9.0	13 (14.1)	
40–49	329	32.3	78 (23.7)	
50–59	394	38.7	87 (22.1)	
≥ 60	204	20.0	33 (16.3)	
Education				0.954
High school or below	868	85.2	161 (20.7)	
Collage or above	151	14.8	50 (20.5)	
Marital status				0.079
Married or cohabitating	728	71.4	2.7 (22.1)	
Unmarried or divorced or widowed	291	28.6	1.3 (17.2)	
Household income, HKD/month				0.177
< 15,000	463	45.4	92 (29.9)	
15,000-30,000	326	32.0	64 (19.6)	
30,000-50,000	151	14.8	31 (20.5)	
≥ 50,000	79	7.8	24 (30.4)	
Employment status				0.472
Full time	378	37.1	77 (20.4)	
Part time	129	12.7	22 (17.1)	
Not working	512	50.2	112 (21.9)	
Menopausal status				0.055
Premenopausal	539	52.9	124 (23.0)	
Postmenopausal	480	47.1	87 (18.1)	
BMI at diagnosis, kg/m^2^				0.007
Underweight (< 18.5)	37	3.6	9 (24.3)	
Normal (18.5–22.9)	488	47.9	108 (22.1)	
Overweight (23–24.9)	209	20.5	54 (25.8)	
Obese (≥25)	285	28.0	40 (14.0)	
Number of comorbidities				0.163
None	629	61.7	144 (22.9)	
1	259	25.4	46 (17.8)	
2	101	9.9	17 (16.8)	
≥ 3	30	2.9	4 (13.3)	
AJCC stage at diagnosis				0.024 ^a^
0-I	375	36.9	61 (16.2)	
II	471	46.2	109 (23.1)	
III	169	16.6	41 (24.3)	
Missing	4	0.4	0 (0)	
Histology				0.899
IDC	858	84.2	176 (20.5)	
ILC	31	3.0	8 (25.8)	
DCIS	56	5.5	11 (19.6)	
Others	74	7.3	16 (21.6)	
ER status				0.969 ^a^
Positive	763	74.9	47 (20.2)	
Negative	233	22.9	159 (20.8)	
Missing	23	2.3	5 (21.7)	
PR status				0.952 ^a^
Positive	598	58.7	81 (20.5)	
Negative	395	38.8	124 (20.7)	
Missing	26	2.6	6 (23.1)	
HER 2 status				0.832 ^a^
Positive	274	26.9	146 (20.1)	
Negative	686	67.3	55 (21.3)	
Missing	59	5.8	10 (16.9)	
Surgery				0.521
Mastectomy	628	61.6	126 (20.1)	
Conservation	391	38.4	85 (21.7)	
Chemotherapy				0.034
Yes	774	76.0	172 (22.2)	
No	245	24.0	39 (15.9)	
Radiotherapy				0.424
Yes	721	70.8	154 (21.4)	
No	298	29.2	57 (19.1)	
Endocrine therapy				0.480
Yes	777	76.3	157 (20.2)	
No	242	23.7	54 (22.3)	
Smoking status				0.133
Yes	11	1.1	0 (0)	
No	1008	98.9	211 (20.9)	
Drinking status				1.00
Yes	11	1.1	3 (20.7)	
No	1008	98.9	208 (20.7)	

^a^ Missing group did not include in *P* value test

*Abbreviations*: *HKD* Hong Kong dollars, *BMI* body mass index, *AJCC* American joint Committee on cancer, *IDC* invasive ductal carcinoma, *ILC* invasive lobular carcinoma, *DCIS* ductal carcinoma in situ, *ER* estrogen receptor, *PR* progesterone receptor, *HER 2* human epidermal-growth-factor receptor 2

Multivariate logistic regression model was used to investigate potential characteristics which could predict higher likelihood of such positive changes. The variables entered into the model were those with *P* < 0.1 in the univariate analysis, which included age at diagnosis, marital status, menopausal status, BMI at diagnosis, AJCC stage and chemotherapy. The results of multivariate analysis are presented in supplementary Table 3. Compared to patients who were aged < 40 years, those who were aged from 40 to 59 years were more likely to make such positive change [age group 40–49 years and 50–59 years: odds ratio (OR) = 2.7, 95%CI: 1.3 ~ 5.8, *P* = 0.008; OR = 3.2, 95%CI: 1.5 ~ 7.2, *P* = 0.004, respectively. Patients who were obese at diagnosis were unlikely to make such change after diagnosis compared to those who were underweight (OR = 0.4, 95%CI: 0.2 ~ 1.0, *P* = 0.042).

### Absolute changes of physical activity between pre- and post-diagnosis by socio-demographic, clinical and lifestyle factors

The absolute changes in level of physical activity between pre- and post-diagnosis by socio-demographic, clinical and lifestyle factors were presented in Table 4. Compared to pre-diagnosis, the level of physical activity increased by 2.2 MET-hours/week at post-diagnosis. Univariate analysis showed that higher increase in physical activity was observed among breast cancer patients aged between 40 and 59 years (compared to those who were aged≥60 years), had a partner (married or cohabitating), not working (compared to full time or part-time) and had no comorbidity (compared to patients who had 1 or more comorbidities).

**Table 4:** Absolute changes in level of physical activity between pre- and post-diagnosis by baseline socio-demographic and clinical factors

Characteristics	N	%	Changes in MET-hours/week, mean ± SD	*P* value
Total	1019	100.0	3.7 ± 12.1	
Age at diagnosis, year				0.011
< 40	92	9.0	4.2 ± 10.8	
40–49	329	32.3	4.8 ± 11.2	
50–59	394	38.7	4.0 ± 12.5	
≥ 60	204	20.0	1.3 ± 13.2	
Education				0.918
High school or below	868	85.2	3.7 ± 12.5	
Collage or above	151	14.8	3.6 ± 9.5	
Marital status				0.006
Married or cohabitating	728	71.4	4.3 ± 12.6	
Unmarried or divorced or widowed	291	28.6	2.2 ± 10.8	
Household income, HKD/month				0.059
< 15,000	463	45.4	3.2 ± 12.9	
15,000-30,000	326	32.0	4.2 ± 11.3	
30,000-50,000	151	14.8	2.6 ± 12.3	
≥ 50,000	79	7.8	6.7 ± 10.4	
Employment status				< 0.001
Full time	378	37.1	2.3 ± 13.4	
Part time	129	12.7	1.7 ± 14.2	
Not working	512	50.2	5.2 ± 10.3	
Menopausal status				0.299
Premenopausal	539	52.9	4.7 ± 11.6	
Postmenopausal	480	47.1	2.6 ± 12.6	
BMI at diagnosis, kg/m^2^				0.086
Underweight (< 18.5)	37	3.6	5.1 ± 13.5	
Normal (18.5–22.9)	488	47.9	4.1 ± 12.3	
Overweight (23–24.9)	209	20.5	4.6 ± 12.1	
Obese (≥25)	285	28.0	2.2 ± 11.6	
Number of comorbidities				0.002
None	629	61.7	4.8 ± 12.0	
1	259	25.4	2.2 ± 12.7	
2	101	9.9	1.2 ± 10.8	
≥ 3	30	2.9	1.3 ± 11.4	
AJCC stage at diagnosis				0.352 ^a^
0-I	375	36.9	3.2 ± 11.7	
II	471	46.2	3.9 ± 13.1	
III	169	16.6	4.4 ± 10.3	
Missing	4	0.4	−5.0 ± 10.3	
Histology				0.437
IDC	858	84.2	3.6 ± 12.2	
ILC	31	3.0	6.4 ± 11.4	
DCIS	56	5.5	2.3 ± 10.0	
Others	74	7.3	4.7 ± 13.3	
ER status				0.394 ^a^
Positive	763	74.9	3.8 ± 11.5	
Negative	233	22.9	3.6 ± 14.0	
Missing	23	2.3	0.3 ± 11.4	
PR status				0.214 ^a^
Positive	598	58.7	4.0 ± 11.8	
Negative	395	38.8	3.5 ± 12.6	
Missing	26	2.6	−0.2 ± 11.8	
HER 2 status				0.124 ^a^
Positive	274	26.9	3.7 ± 12.8	
Negative	686	67.3	4.0 ± 12.1	
Missing	59	5.8	0.6 ± 9.4	
Surgery				0.648
Mastectomy	628	61.6	3.8 ± 13.1	
Conservation	391	38.4	3.5 ± 10.4	
Chemotherapy				0.127
Yes	774	76.0	4.0 ± 12.7	
No	245	24.0	2.7 ± 10.1	
Radiotherapy				0.944
Yes	721	70.8	3.7 ± 11.7	
No	298	29.2	3.7 ± 13.2	
Endocrine therapy				0.789
Yes	777	76.3	3.6 ± 11.2	
No	242	23.7	3.9 ± 14.6	
Smoking status				0.455
Yes	11	1.1	1.0 ± 3.7	
No	1008	98.9	3.7 ± 12.2	
Drinking status				0.866
Yes	15	1.5	4.2 ± 11.6	
No	1004	98.5	3.7 ± 12.1	

^a^ Missing group did not include in *P* value test

*Abbreviations*: *MET* metabolic equivalent of task, *SD* standard deviation, *HKD* Hong Kong dollars, *BMI* body mass index, *AJCC* American joint Committee on cancer, *IDC* invasive ductal carcinoma, *ILC* invasive lobular carcinoma, *DCIS* ductal carcinoma in situ, *ER* estrogen receptor, *PR* progesterone receptor, *HER 2* human epidermal-growth-factor receptor 2

Multivariate linear regression model was used to investigate the association between absolute changes in level of physical activity and socio-demographic, clinical and lifestyle factors. The variables entered into the model were those with *P* < 0.1 in the univariate analysis, which included age at diagnosis, marital status, household income, employment status, BMI at diagnosis and number of comorbidities. The results are presented in supplementary Table 4. Compared to patients who had a partner (married or cohabitating), those who were single (unmarried, divorced or widowed) had lower increase in level of physical activity between pre and post-diagnosis (β = − 2.0, 95%CI: − 3.7 ~ − 0.3, *P* = 0.021). Patients who were not working at baseline showed more increase in level of physical activity after diagnosis compared to those in full-time job (β = 2.7, 95%CI: 0.9 ~ 4.5, *P* = 0.002). Patients with one comorbidity at baseline showed lower increase in level of physical activity after diagnosis compared with those who had no comorbidity (β = − 2.8, 95%CI: − 4.7 ~ − 1.0, *P* = 0.002).

## Discussion

This is the first prospective cohort study that compared the level of physical activity among Chinese breast cancer survivors from 1 year before cancer diagnosis to 5 years after diagnosis. Compared to pre-diagnosis, patients significantly increased their level of physical activity at 18-months post-diagnosis, and the enhanced level of physical activity was well-sustained at 36-months and 60-months post-diagnosis. When individual change in physical activity was assessed, the majority of patients improved or had no change in their level of physical activity at post-diagnosis. This suggests that the diagnosis of cancer provided an opportune time to motivate individuals to adopt healthier lifestyle changes, including physical activity [17, 18]. In this study, we see evidence that a cancer diagnosis was associated with sustained improvement in physical activity for at least 5 years after diagnosis. However, the proportion of women that met the exercise recommendations was still relatively low after cancer diagnosis. Additionally, multivariate analysis showed that marital status, employment status and comorbidities were associated with significant magnitude of change in level of physical activity between pre- and post-diagnosis.

There have been several qualitative studies, which showed that between 16 and 32% of patients increased their exercise level after diagnosis [33–35]. The proportion of patients who increased exercise after diagnosis was relatively higher in this study, at 48.2%. In addition, a few prospective studies had quantitatively compared the physical activity level before and after breast cancer diagnosis in Western population, but the majority of these studies compared physical activity level during a relative short time-window, for instance, from the year before breast cancer diagnosis to 6 or 12-month following cancer diagnosis [19, 21–23, 36]. These studies suggested that compared to pre-diagnosis, patients significantly decreased their level of exercise during active adjuvant treatment. One the other hand, in a German study that included 229 breast cancer patients, the change in physical activity before diagnosis and 14 months after diagnosis was assessed. The investigators reported that 46.1% of the patients spent more time on physical activity after diagnosis, with an increase from 2.2 to 2.9 h/week. With a more protracted follow-up, findings from the present study demonstrated that the level of physical activity was higher at 18-month follow-up compared to pre-diagnosis of cancer, and such increase was sustained at 36-months and 60-months follow-up. Up to now, only one study has investigated the change of physical activity over a more protracted period, from 2-year before diagnosis to 30-month after diagnosis, and this was conducted among women with breast cancer in the US [25]. Relative to pre-diagnosis, the average level of physical activity decreased by 50% in the first 12 months after diagnosis; this gradually recovered at 19–30 months after diagnosis, but still remained about 3 MET-hours/week lower than the level at pre-diagnosis [25]. Possible reasons for the differences observed between the aforementioned study and the current study may be related to different level of physical activity at pre-diagnosis. The mean level of physical activity ranges from 13 to 18.8 MET-hours/week in the US and Belgium studies, [22, 25] which was higher than the corresponding figure in the present study (median value of 0.6 MET-hours/week). Taken together, all these evidences suggested that during short-term follow-up, patients who were still on active adjuvant treatment did not increase their level of physical activity. As follow-up time became longer, patients would have completed most adjuvant treatment (except for endocrine therapy) and this paralleled the gradually increase in their level of physical activity.

Several studies have examined the factors which could predict the changes of physical activity before and after cancer diagnosis [19, 23, 25]. The association between BMI and changes in physical activity has not been well-defined: two studies reported that obese and overweight women had greater decrease in physical activity after diagnosis relative to normal weight women, [19, 23, 25] while another study showed that patients who were underweight or had normal BMI had greater reductions in physical activity.^50^ This study showed that patients with higher BMI had a trend in lower increase of physical activity at post-diagnosis. On the other hand, obese women were unlikely to change into high level of physical activity compared to underweight women; this suggests that healthcare professionals may need to pay more attention in counselling overweight and obese patients. Results from multivariate analysis in the present study found that women in relationship had a higher increase in the level of physical activity compared with women who were not in relationship. This phenomenon has also been reported in a previous study, which suggested that marital status was an important determinant for physical activity participation among older adults [37]. In addition, patients who were not employed had higher increase in physical activity than full-time working women, which may be explained by non-working ones having more leisure time. Furthermore, in support to the present finding, Troeschel et al. have reported that each additional comorbidity was associated with a 26% increase in odds of inactivity [38]..

In recent decades, the improvement in cancer diagnostics, treatment and clinical care have contributed to improved survival of breast cancer patients. However, at the same frame, patients could face long-term side effects of treatment and hence they have various rehabilitation needs. A systematic review which used comprehensive approach to retrieve literature and aimed to investigate the effects of rehabilitation interventions in post-treatment breast cancer patients, supported the benefit of exercise interventions, and showed that exercise could improve outcomes such as shoulder mobility, lymphoedema, pain, fatigue and quality of life [39]. Additionally, physical activity also have several other well-known benefits, including prevention of chronic disease and weight management [40]. These benefits are important for breast cancer survivors who are at higher risk of developing second primary cancer, other chronic illnesses such as cardiovascular disease and diabetes, as well as facing psychological stress of living with a diagnosis of cancer [41–45]. Furthermore, increasing evidence have also shown that physical activity is associated with reduced risk of recurrence and better prognosis for breast cancer [46–53].

The present study has shown that the level of physical activity was significantly increased up to 60-months post-diagnosis compared to that of pre-diagnosis, while no significant difference was noted between the follow-up assessments at 18-, 36- and 60-months post-diagnosis. This suggested that breast cancer survivors adopted increased physical activity soon after their cancer diagnosis, and such changes were sustained as time went on. As increasing evidence have shown that physical activity is associated with better long-term outcome [46–53] and quality of life [54] in women with breast cancer, healthcare professionals should grasp this important window of opportunity to encourage continued and enhanced physical activity. Follow-up of this cohort would provide important information on whether improved physical activity is associated with health outcomes among Chinese breast cancer survivors. However, it has to be noted that although most of the patients became more physically active during follow-ups, they still did not reach the recommended level of physical activity.

The strengths of the present study included its large, prospective design, based on the data collected from validated modified Chinese Baecke questionnaire that included measurement of physical activity in doing sports. Furthermore, this study compared physical activity between pre-diagnosis with three time-points after cancer diagnosis, which allows the investigators to understand the relative long-term changes made among breast cancer patients. There are several limitations in this study. Firstly, physical activity was based on self-reported questionnaire, thus overestimation or underestimation was possible. Nevertheless, this potential bias was reduced by the application of the same questionnaire over the four time-points. Objective instruments, such as accelerometer, would be more useful in future studies to monitor physical activity in a more accurate manner. Secondly, the modified Chinese Baeke questionnaire included physical activity at work, in doing housework, at leisure time and in doing sports. However, the present study only analyzed the level of physical activity in doing sports. Additionally, the modified Chinese Baecke questionnaire has only been validated in a random sample of Hong Kong Chinese adult population, although not specifically validated in cancer patients. Thirdly, although the majority of the participants included in the current study had stable or improvement in level of physical activity, these findings may not be able to represent the whole breast cancer population in Hong Kong. The present findings should be interpreted with care because there may be a potential selection bias, as patients who were more concerned with health improvement would be those who were more likely to agree to participate in this study. In addition, as sizeable number of patients (*n* = 443) in the whole cohort were not included in this analysis, it may affect the generalization of study results. Moreover, the loss of subjects may contribute to selection bias if those lost to follow up are more or less likely to perform exercise. Fourthly, it has to be acknowledged that the sample size was not estimated for the present analysis. Last but not least, given this study was undertaken in two regional cancer centers, the patients might have adopted different potential lifestyle interventions based on education, counseling, sports groups that were offered in individual center. Hence, the lack of standardization of lifestyle educational intervention could affect the study results.

## Conclusions

The present findings provide important evidence on breast cancer patients’ habits on sports activity following their diagnosis. In the present study, significant and long-term increase in post-diagnosis sports activity were observed among Chinese women with breast cancer, which was accord with current lifestyle recommendation from international authorities. However, the proportion of patients who met the recommended exercise level for cancer survivors was still low. These findings provided an insight into the fact that a cancer diagnosis can motivate patients to adopt a healthy lifestyle. Encouraging patients on the importance of durable high level of physical activity in breast cancer survivorship is warranted. In addition, long-term follow-up is needed to provide more evidence on possible association of high physical activity and cancer outcome.

## Supplementary information

**Additional file 1 Supplementary Table 1**. Modified Chinese Baecke Questionnaire. **Supplementary Table 2**. Changes in level of physical activity between pre- and post-Changes in level of physical activity between pre- and post-diagnosis for individual patient. **Supplementary Table 3**. Multivariable logistic regression model assessing changes from no or low to high level of exercise group for individual patient in relation to socio-demographic and clinical factors. **Supplementary Table 4**. Multivariable linear regression model assessing absolute changes in level of physical activity between pre- and post-diagnosis in relation to socio-demographic and clinical factors.

## Data Availability

All analyzed data during the current study were presented in the main manuscript and supplementary file. The original datasets are available from the corresponding author on reasonable request.

## Abbreviations

BMI: Body mass index
ACS: American Cancer Society
WCRF/AICR: World Cancer Research Fund/American Institute for Cancer Research
MET: Equivalent of task
BCRL: Breast cancer related lymphedema
AWS: Axillary web syndrome
SD: Standard deviation
HKD: Hong Kong dollars
AJCC: American joint Committee on cancer
IDC: Invasive ductal carcinoma
ILC: Invasive lobular carcinoma
DCIS: Ductal carcinoma in situ
ER: Estrogen receptor
PR: Progesterone receptor
HER 2: Human epidermal-growth-factor receptor 2
