# Supplementary material for: Longitudinal changes in sports activity from pre-diagnosis to first five years post-diagnosis: a prospective Chinese breast cancer cohort study
Source: BMC Cancer. 2020 Oct 19;20:1013. doi: 10.1186/s12885-020-07517-6 (PMC7574482; doi:10.1186/s12885-020-07517-6)
Supplement: Supplementary file 1 — Additional file 1 Supplementary Table 1. Modified Chinese Baecke Questionnaire. Supplementary Table 2. Changes in level of physical activity between pre- and post-Changes in level of physical activity between pre- and post-diagnosis for individual patient. Supplementary Table 3. Multivariable logistic regression model assessing changes from no or low to high level of exercise group for individual patient in relation to socio-demographic and clinical factors. Supplementary Table 4. Multivariable linear regression model assessing absolute changes in level of physical activity between pre- and post-diagnosis in relation to socio-demographic and clinical factors. [file 12885_2020_7517_MOESM1_ESM.docx]

Supplementary Tables.

Supplementary Table 1. Modified Chinese Baecke Questionnaire

| **Work Index** | | |
| --- | --- | --- |
| 1. What is your main occupation? . . . |  | 1-3-5 |
| 2. Do you often sit at work? | Never/seldom/sometimes/always | 4 - 3 - 2 - 1 |
| 3. Do you often stand at work? | Never/seldom/sometimes/always | 1 - 2 - 3 - 4 |
| 4. Do you often walk at work? | Never/seldom/sometimes/always | 1 - 2 - 3 - 4 |
| 5. Do you often lift heavy loads at work? | Never/seldom/sometimes/always | 1 - 2 - 3 - 4 |
| 6. Do you often feel physically tired after working? | Never/seldom/sometimes/always | 1 - 2 - 3 - 4 |
| 7. Do you often sweat at work? | Never/seldom/sometimes/always | 1 - 2 - 3 - 4 |
| 8. When compared with people of your own age and sex, do you think your work is physically much heavier or lighter? | Lighter/as heavy/heavier | 1 - 2 - 3 |
| **Sport Index** | | |
| 9. Do you play sport when you don’t need to work or do household activities? Yes/No  If yes: –which sport do you play most frequently? . . .  –how many hours a week?  –how many months a year?  If you play a second sport:  –which sport is it? . . .  –how many hours a week?  –how many months a year? | 1 / 3 / 5  0/<1/1-2/2-3/3-4/>4  0/<1/1-3/4-6/7-9/>9  1 / 3 / 5  0/<1/1-2/2-3/3-4/>4  0/<1/1-3/4-6/7-9/>9 | 0.76-1.26-1.76  0-0.5-1.5-2.5-3.5-4.5  0-0.04-0.17-0.42-0.67-0.92  0.76-1.26-1.76  0-0.5-1.5-2.5-3.5-4.5  0-0.04-0.17-0.42-0.67-0.92 |
| 10. In comparison with others of my own age I think my physical activity during leisure time is . . . | Less/the same/more | 1 - 2 - 3 |
| 11. Do you play sport during leisure time? | Never/seldom/sometimes/often | 1 - 2 - 3 - 4 |
| **Leisure Time Index** | | |
| 12. Do you often watch television/video/laser disc/see movie during leisure time? | Never/seldom/sometimes/always | 4 - 3 - 2 - 1 |
| 13. Do you often walk for pleasure during leisure time? | Never/seldom/sometimes/always | 1 - 2 - 3 - 4 |
| 14. How many minutes per day do you walk and/or cycle to and from work, school, and shopping? | <10/10-30/30-60/60-120/>120 | 1 - 2 - 3 - 4 - 5 |
| 15. Do you often go shopping (not including grocery shopping) during leisure time? | Never/seldom/sometimes/often/very often | 1 - 2 - 3 - 4 |
| 16. Do you often play mahjong during leisure time? | Never/seldom/sometimes/often/very often | 4 - 3 - 2 - 1 |
| **Housework Index** | | |
| 17. Do you often do heavy household work (e.g., extensive clean-up, washing floors, washing windows, washing toilet, washing kitchen, cleaning all furniture and electrical appliances, hanging wet linens, etc.)? | Never/seldom/sometimes/often | 1 - 2 - 3 - 4 |
| 18. Do you often do light household work (e.g., general clean-up, dusting, vacuuming, sweeping floor, handwashing clothes, hanging clothes, ironing, hanging linen, etc.)? | Never/seldom/sometimes/often | 1 - 2 - 3 - 4 |
| 19. Do you often prepare meals? | Never/seldom/sometimes/often | 1 - 2 - 3 - 4 |
| 20. Do you often wash dishes? | Never/seldom/sometimes/often | 1 - 2 - 3 - 4 |
| 21. Do you often do grocery shopping? | Never/seldom/sometimes/often | 1 - 2 - 3 - 4 |
| 22. How many days per week do you go to the market? | 1-2/3-4/5-7 | 1 - 2 - 3 |
| 23. How many minutes/hours per day on average do you walk to and from grocery shopping (not including transportation time)?  (If not doing grocery shopping every day, write down on average how many days per week he/she does it.) | <10/10-30/30-60/60-120/>120 | 1-2-3-4-5 |
| 24. How many hours per day do you sleep on average? | <7/7+ hours | 1 - 1.5 |
| Calculation of the score for question 9  If type of sport = 1, then Intensity = 0.76  If type of sport = 3, then Intensity = 1.26  If type of sport = 5, then Intensity = 1.76  If number of hours a week = <1 then Time = 0.5  If number of hours a week = 1-2 then Time = 1.5  If number of hours a week = 2-3 then Time = 2.5  If number of hours a week = 3-4 then Time = 3.5  If number of hours a week = >4 then Time = 4.5  If number of months a year = <1 then Proportion = 0.04  If number of months a year = 1-3 then Proportion = 0.17  If number of months a year = 4-6 then Proportion = 0.42  If number of months a year = 7-9 then Proportion = 0.67  If number of months a year = >9 then Proportion = 0.92  Question 9 = (Intensity1 * Time1 * Proportion1)  + (Intensity2 * Time2 * Proportion2) | 0/0.01-<4/4-<8/8-<12/≥12*  *0 is given to people who do not play sport. | 1 - 2 - 3 - 4 - 5 |
| Calculation of physical activity indices  The subscript for I corresponds to the question item number.  WORK INDEX (WI) = (I1 +I2 + I3 + I4 + I5 +I6 +I7 + I8)/8  SPORTS INDEX (SI) = (I9 +I10 + I11)/3  LEISURE TIME INDEX (LTI) = (I12 + I13 + I14 +I15 +I16)/5  HOUSEWORK INDEX (HWI) = (I17+I18+I19+I20+I21+I22+I23)/7  TOTAL INDEX (TI) = = WI + SI + LTI + HWI | | |

Supplementary Table 2. Changes in level of physical activity between pre- and post-Changes in level of physical activity between pre- and post-diagnosis for individual patient

| Type of change | N | Frequency, % |
| --- | --- | --- |
| Improved  No 🡪 Low  No🡪 High  Low 🡪 High | **491**  280  106  105 | **48.2**  27.5  10.4  10.3 |
| No change  No 🡪 No  Low 🡪 Low  High 🡪 High | **446**  87  212  147 | **43.8**  8.6  20.8  14.4 |
| Declined  Low 🡪 No  High 🡪 Low  High 🡪 No | **82**  18  58  6 | **8.0**  1.7  5.7  0.6 |

Supplementary Table 3. Multivariable logistic regression model assessing changes from no or low to high level of exercise group for individual patient in relation to socio-demographic and clinical factors

| Characteristics | OR | 95%CI | *P* value |
| --- | --- | --- | --- |
| Age at diagnosis, year (ref: <40)  40-49  50-59  ≥60 | 2.7  3.2  2.4 | 1.3~5.8  1.5~7.2  0.9~6.2 | 0.008  0.004  0.062 |
| Marital status (ref: Married or cohabitating)  Unmarried or divorced or widowed | 0.8 | 0.5~1.1 | 0.148 |
| Menopausal status (ref: Premenopausal)  Postmenopausal | 0.7 | 0.4~1.1 | 0. 082 |
| BMI at diagnosis, kg/m^2^(ref: <18.5)  Normal (18.5-22.9)  Overweight (23-24.9)  Obese (≥25) | 0.8  0.8  0.4 | 0.4~1.8  0.4~2.0  0.2~1.0 | 0.476  0.652  0.042 |
| AJCC stage at diagnosis (ref: 0-I)  II  III | 1.6  1.7 | 1.0~2.5  0.9~2.9 | 0.051  0.067 |
| Chemotherapy (ref: No)  Yes | 1.2 | 0.7~2.0 | 0.437 |

Abbreviations: OR, odds ratio; CI, confidential interval; ref, reference; BMI, body mass index; AJCC, American joint Committee on cancer.

Supplementary Table 4. Multivariable linear regression model assessing absolute changes in level of physical activity between pre- and post-diagnosis in relation to socio-demographic and clinical factors

| Characteristics | β | 95%CI | *P* value |
| --- | --- | --- | --- |
| Age at diagnosis, year (ref: <40)  40-49  50-59  ≥60 | 1.1  1.7  -0.2 | -1.7~4.0  -1.2~4.6  -3.5~3.1 | 0.443  0.248  0.896 |
| Marital status (ref: Married or cohabitating)  Unmarried or divorced or widowed | -2.0 | -3.7~-0.3 | 0.021 |
| Household income, HKD/month (ref: <15,000)  15,000-30,000  30,000-50,000  ≥50,000 | -0.5  -2.3  1.2 | -2.3~1.3  -4.6~0.1  -1.9~4.2 | 0.567  0.052  0.461 |
| Employment status (ref: Full time)  Part time  Not working | -1.4  2.7 | -4.0~1.0  0.9~4.5 | 0.242  0.002 |
| BMI at diagnosis, kg/m^2^(ref: <18.5)  Normal (18.5-22.9)  Overweight (23-24.9)  Obese (≥25) | -0.8  -0.2  -2.0 | -4.8~3.1  -4.3~4.1  -6.1~2.1 | 0.682  0.961  0.333 |
| Number of comorbidities (ref: 0)  1  2  ≥3 | -2.8  -2.1  -2.6 | -4.7~-1.0  -4.8~0.7  -6.2~2.7 | 0.002  0.143  0.437 |

Abbreviations: CI, confidential interval; ref, reference; HKD, Hong Kong dollars; BMI, body mass index.
